# Glycerol kinase 5 confers gefitinib resistance through SREBP1/SCD1 signaling pathway

**DOI:** 10.1186/s13046-019-1057-7

**Published:** 2019-02-21

**Authors:** Jian Zhou, Guimei Qu, Ge Zhang, Zuoren Wu, Jing Liu, Dawei Yang, Jing Li, Meijia Chang, Hengshan Zeng, Jie Hu, Tao Fang, Yuanlin Song, Chunxue Bai

**Affiliations:** 1 Department of Pulmonary Medicine, Shanghai Respiratory Research Institute, Zhongshan Hospital, Fudan University, Shanghai, China; 2 Department of Pathology, The Affiliated Yantai Yuhuangding Hospital, Qingdao University, Yantai, China; 3 Hangzhou Dixiang Co. Ltd., Hangzhou, China; 4 Department of Oncology, Shengli Oilfield Central Hospital, Dongying, Shandong Province China

**Keywords:** Non-small cell lung cancer, Glycerol kinase 5, Gefitinib, Stearoyl-CoA desaturase-1

## Abstract

**Background:**

Drug resistance is common in cancer chemotherapy. This study investigates the role of Glycerol kinase 5 (GK5) in mediating gefitinib resistance in NSCLC.

**Methods:**

The exosomal mRNA of GK5 was detected using a tethered cationic lipoplex nanoparticle (TCLN) biochip. Real-time PCR and Western blot were used to examine the expression of GK5 mRNA and protein in gefitinib-sensitive and -resistant human lung adenocarcinoma cells. The cell counting kit-8, EdU assay, flow cytometry, and JC-1 dye were used to measure cell proliferation, cell cycle, and the mitochondrial membrane potential.

**Results:**

We found that the exosomal mRNA of GK5 in the plasma of patients with gefitinib-resistant adenocarcinoma was significantly higher compared with that of gefitinib-sensitive patients. The mRNA and protein levels of GK5 were significantly upregulated in gefitinib-resistant human lung adenocarcinoma PC9R and H1975 cells compared with gefitinib-sensitive PC9 cells. Silencing GK5 in PC9R cells induced mitochondrial damage, caspase activation, cell cycle arrest, and apoptosis via SREBP1/SCD1 signaling pathway.

**Conclusions:**

We demonstrated that GK5 confers gefitinib resistance in lung cancer by inhibiting apoptosis and cell cycle arrest. GK5 could be a novel therapeutic target for treatment of NSCLC with resistance to EGFR tyrosine kinase inhibitors.

## Background

Lung cancer is one of the most common malignancies and is the leading cause of cancer-related death worldwide [1]. About 80% of lung cancer is non-small cell lung cancer (NSCLC). Mutation of the epidermal growth factor receptor (EGFR) gene is one of the common driving causes of NSCLC [2, 3]. The frequency of EGFR gene mutation is as high as 60% in Asian non-smoking patients. EGFR tyrosine kinase inhibitors (TKIs) are the important targeted drug for treating such NSCLC [4, 5]. However, NSCLC patients eventually develop resistance to TKIs [6, 7]. Secondary EGFR mutations including Thr790Met and MET gene amplification are the major mechanisms of resistance. There are about 20–30% of NSCLC patients with unknown mechanisms of resistance [8, 9]. Therefore, it is critical to clarify new signaling pathways involved in EGFR-TKI resistance.

Lipid metabolism such as fatty acid, phospholipid and triacylglycerol synthesis plays an important role in cancer progression by maintaining cellular structure, providing energy and signaling molecules [10]. Sterol regulatory element-binding protein 1 (SREBP1) is a critical transcription factor, and is overexpressed in various cancers and promotes cell proliferation, invasion, and migration [11–16]. SREBP1 is synthesized as a 125 kDa precursor, which is cleaved into the 65 kDa mature activating enzyme [15, 16]. Stearoyl-CoA-desaturase 1 (SCD1) is an enzyme involved in lipid metabolism. It converts palmitic and stearic acids to mono-unsaturated fatty acids, a critical step shifting fatty acid oxidation to lipogenesis. SCD1 has been demonstrated to be overexpressed in various cancers including lung cancer, and increases cancer initiation, survival and invasiveness, leading to poor patient prognosis [17–22].

EGFR is overexpressed in many types of cancers, and activates various downstream signalling pathways including the Phosphoinositide 3-kinase/Akt pathway [23], which activates SREBP1 cleavage and up-regulates SCD1, acetyl-coa carboxylase (ACC), and fatty acid synthase (FASN), leading to enhanced lipid metabolism [13, 22]. EGFR has tyrosine kinase independent functions, that are important for cell proliferation, because EGFR silencing decreases phosphorylated AKT (p-AKT), phosphorylated extracellular signal-regulated kinase (p-ERK) and cell apoptosis [24–29]. Furthermore, EGFR has been demonstrated to modulate glucose level in cancer cells by regulating sodium/glucose cotransporter 1 (SGLT1) independent of receptor tyrosine kinase activities [29].

Glycerol kinase (GK) is a rate-limiting enzyme converting glycerol to glycerol 3-phosphate [30], which links glycolysis and lipid metabolism [10]. Reduction of GK activity significantly decreases glycerolipids [31]. GK has alternative functions causing insulin resistance, apoptosis, and cell cycle arrest [32–34]. GK knockout mice leads to neonatal death after birth [35]. There are three types of GKs including GK, GK2, and GK5 [36]. The function of GK5 in EGFR-TKI resistance has not been studied.

In this study, we found that GK5 is upregulated in specimens of lung cancer resistant to EGFR-TKIs. GK5 promotes gefitinib resistance by inhibiting apoptosis and cell cycle arrest. Knockdown of GK5 in gefitinib-resistant cells restores sensitivity through repressing SCD1 signal pathway. Our results suggested that GK5 could be a mediator of resistance to EGFR tyrosine kinase inhibitors.

## Materials and methods

### Detecting exosomal GK5 mRNA

This study was approved by the Research Ethics Committee of Zhongshan Hospital, Fudan University (Shanghai, China) and performed according to relevant guidelines and regulations. Written informed consent was obtained from all participating individuals. EDTA plasma samples from 17 individuals with lung adenocarcinoma, who were sensitive to EGFR TKIs, and 11 individuals with lung adenocarcinoma, who had acquired resistance to EGFR TKIs, admitted at the Department of Pulmonary Medicine, Zhongshan Hospital, Fudan University. The Invitrogen total exosome precipitation reagent (Thermo Fisher Scientific, MA, USA) was used to isolate the exosomes from plasma samples according to manufacturer’s instruction. The detection of exosomal GK5 mRNA, using tethered cationic lipoplex nanoparticles (TCLNs), was previously described [37, 38]. Cationic lipoplex nanoparticles, containing the GK5 molecular beacons (MBs, custom synthesized by Sigma-Aldrich, MO, USA), were tethered onto the glass slide surface by a biotin-avidin linkage. All the MBs were labeled with Fluorescein amidite (FAM). To detect the expression of GK5 mRNA in the exosomes, Total Internal Reflection Fluorescence (TIRF) Microscopy (Nikon, Japan) was used. A custom-developed MATLAB program was applied to analyze the images by generating a binary mask to remove the background and measure the sum intensity of each image. In this study, PBS controls were used to define the background. Any fluorescence signals from the samples that were equal or lower than the signals observed in the PBS controls were defined as background in the image analysis.

### Cell culture

The human lung adenocarcinoma cell line PC9 and NCI-H1975 (H1975, intrinsically harbor EGFR L858R/T790 M) were purchased from the American Type Culture Collection (ATCC, Rockville, MD, USA). To induce gefitinib resistance, PC9 cells were exposed to increasing concentrations of gefitinib [39]. The gefitinib-sensitive PC9 and -resistance PC9R and H1975 cells were cultured in RPMI 1640 medium (Thermo Fisher Scientific, MA, USA) supplemented with 10% heat-inactivated fetal bovine serum and 100 U/ml penicillin/streptomycin. The gefitinib resistance in the PC9R and H1975 cells was maintained by adding 1 μΜ gefitinib (Selleckchem, TX, USA). The cells were grown as monolayers in a humidified atmosphere containing 5% CO_2_ at 37 °C.

### Lentiviral construction and infection

Short hairpin RNA (shRNA) vectors against the GK5 genes shGK5–1 and shGK5–2, and against the SCD1 genes shSCD1 were obtained from TRC (The RNAi Consortium). Lentiviral plasmids, containing GV112-shGK5–1, -shGK5–2, -shSCD1, and -negative, were obtained from GeneChem (Shanghai, China). Lentiviruses carrying overexpressing human EGFR (GenBank accession number NM_005228) lentiviral vectors (GV358) were from GeneChem. Lentiviruses carrying overexpressing human GK5 (GenBank accession number NM_001039547.2) and SCD1 (GenBank accession number AB032261.1) lentiviral vectors (Lv105) were from Genecopoeia (Guangzhou, China). The lentiviral particles were produced by transfecting the HEK 293 T cells with the lentiviral plasmids. For viral infection, PC9R or H1975 cells were plated in 6-well plates, grown to 50–70% confluence, incubated with medium containing virus and 4 μg/mL polybrene at an MOI (multiplicity of infection) of 10, and were then incubated with various concentrations of gefitinib after 24 h.

### Cell proliferation and viability assay

The Cell Counting Kit-8 (CCK-8; Dojindo Laboratories, Japan) was used to assess the rate of cell proliferation. Briefly, cells were plated in 96-well plates at approximately 1000 cells per well with 200 μL of culture medium. After 24 h, 10 μl of CCK8 solution was applied to each well, and the plates were incubated for 1 h at 37 °C. Finally, the absorbance values at 450 nm were determined using a microplate reader (Multiskan, Thermo Fisher Scientific, MA, USA) with a reference wavelength of 650 nm. All experiments were conducted at least in triplicate.

### EdU incorporation assay

Cells were incubated with 10 μM EdU (5-ethynyl-2′-deoxyuridine, Thermo Fisher Scientific, MA, USA) for 4 h and were then fixed with 3.7% formaldehyde in PBS for 15 min at room temperature. The EdU was detected for EdU incorporation according to manufacturer’s recommendations. Confocal imaging was performed on a Nikon A1R confocal laser scanning microscope system (Nikon Corp., Tokyo, Japan). PC9R cells positive for EdU incorporation and positive for Hoechst 33342 staining were counted using Image J (v. 1.42, Wayne Rasband, NIH) and were used to calculate the percentage of EdU-positive cells.

### Detecting apoptosis by flow cytometry

An annexin-V-allophycocyanin (APC) and 4′,6-diamidino-2-phenylindole (DAPI) double staining kit (Thermo Fisher Scientific) was used to evaluate apoptosis. The transfected PC9R cells were seeded in 6-well plates (5 × 10^5^ cells/well) and were treated with 1 μM gefitinib. Cells were then digested with trypsin (Gibco® Trypsin-EDTA, Thermo Fisher Scientific, MA, US), washed with PBS three times, suspended in 500 μl of binding buffer, and were then incubated with 5 μl APC-conjugated annexin-V and 3 μl DAPI for 15 min at room temperature in the dark. The stained cells were detected using the BD FACS Aria II flow cytometer (BD biosciences, CA, USA).

### Cell cycle analysis

The transfected PC9R cells were seeded in 6-well plates (5 × 10^5^ cells/well) and were treated with 1 μM gefitinib. The treated cells were then collected, washed with PBS, and fixed in 70% ethanol for 24 h at 4 °C. The fixed cells were stained with PI in the dark for 30 min at room temperature. Finally, cell cycle distribution was analyzed by flow cytometry.

### Mitochondrial membrane potential measurement

The MitoProbe™ JC*-*1 assay kit (Thermo Fisher Scientific) was used to detect changes in the mitochondrial membrane potential. The assay was performed according to the manufacturer’s instructions. The results were obtained by BD FACS Aria II flow cytometer. JC-1 forms J-aggregates emitting red fluorescence at 590 nm in healthy mitochondria and J-monomers emitting green fluorescence at 490 nm in depolarized mitochondria. An increased ratio of J-monomers indicates mitochondrial damage.

### Quantitative RT-PCR

Total RNA was extracted using the TRIzol reagent (Thermo Fisher Scientific, MA, USA), and cDNA was synthesized using reverse transcriptase (TOYOBO, Japan). The RNA (1%) was reverse transcribed into complementary deoxyribonucleic acid, and 20 ng of complementary DNA was used as the template for RT-PCR. The amplification cycling parameters (40 cycles) were set as follows: 15 s at 95 °C; 15 s at 60 °C and 45 s at 72 °C. The primer sequences included the following:

GK5 sense 5’-TGAGGGACACAAGCCACAAT-3′,

GK5 anti-sense 5’-GGAAGCAGCACTCTCCAAAC-3′.

SCD1 sense 5’-GTACCGCTGGCACATCAACTT-3′,

SCD1 anti-sense 5’-TTGGAGACTTTCTTCCGGTCAT-3′.

β-actin sense 5’-CTGGCACCCAGCACAATG-3′,

β-actin anti-sense 5’-CCGATCCACACGGAGTACTTG-3′.

The gene expressions were normalized to β-actin and were measured by 2^-ΔΔCT^. The RT-PCR assay was performed at least 3 separate times in triplicate.

### Western blot assay

Total protein was extracted using a RIPA kit (Beyotime Biotechnology, China), separated on polyacrylamide gels, and transferred to PVDF membranes. The membranes were incubated with anti-GK5 (Proteintech Group, IL, USA), anti-EGFR (Cell Signaling Technology (CST), MA, USA), anti-survivin (CST), anti-PLK1 (CST), anti-Bcl2 (CST), anti- cleaved caspase-3 (Asp175, CST), anti-caspase-3 (CST), anti- cleaved PARP (Asp214, CST), anti-PARP (CST), anti-SCD1 (Abcam, MA, USA), anti-SREBP1 (Abcam), and anti-actin (CST) antibodies at 4 °C overnight and were then incubated with horseradish peroxidase-conjugated goat anti-rabbit or anti-mouse immunoglobulin G at room temperature for 1 h. Proteins were visualized using Pierce ECL Western blotting substrate and autoradiography. The blots were analyzed using Quantity One 4.6.

### cDNA array and pathway analysis

Total RNA was extracted from the negative control cells and the PC9R cells transfected with shGK5–1 using the RNeasy Plus Mini Kit (Qiagen, MD, USA). The extracted RNA was converted to double-stranded cDNA and was amplified using a OneArray plus RNA amplification kit (Phalanx Biotech Group, Taiwan). The Cy5-labeled RNA targets were hybridized to the Human Whole Genome OneArray (Phalanx Biotech Group, Taiwan). The intensity of signals was measured by the Agilent Microarray Scanner (G2505C, Agilent, USA) and was analyzed by the Rosetta Resolver System (Rosetta Biosoftware). Testing was performed in triplicate, and the statistical analyses were performed using the proprietary modeling techniques from the Rosetta Resolver.

### Tumorigenicity assay

The animal experiments were approved by the Institutional Animal Care and Use Committee at Zhongshan Hospital from Fudan University, China. Twenty four- to 6-week-old male BALB/c nude mice were obtained from the Shanghai Experimental Animal Center of the Chinese Academy of Sciences (Shanghai, China). 1 × 10^7^ PC9R cells transfected with shNEG or shGK5–1 were subcutaneously injected into the right flanks of ten nude mice. The tumor volume was monitored and was calculated according to the following formula: Volume = length x width^2^/2. After one month, tumors were dissected out and weighed for quantification. Ki67 immunohistochemistry staining was applied to identify the proliferating cells in the paraffin sections of the xenograft tumors. The Ki67-positive cells were quantified in randomly selected fields from each tissue section using Image J.

### Statistical analysis

Data are expressed as the means ± SD of at least three independent experiments. Statistical analysis was performed using a one-way analysis of variance (ANOVA) followed by Bonferroni’s multiple comparison test. A *p*-value of < 0.05 was considered statistically significant.

## Results

### GK5 expression is upregulated in gefitinib-resistant patients and is associated with gefitinib resistance

**Fig. 1:**
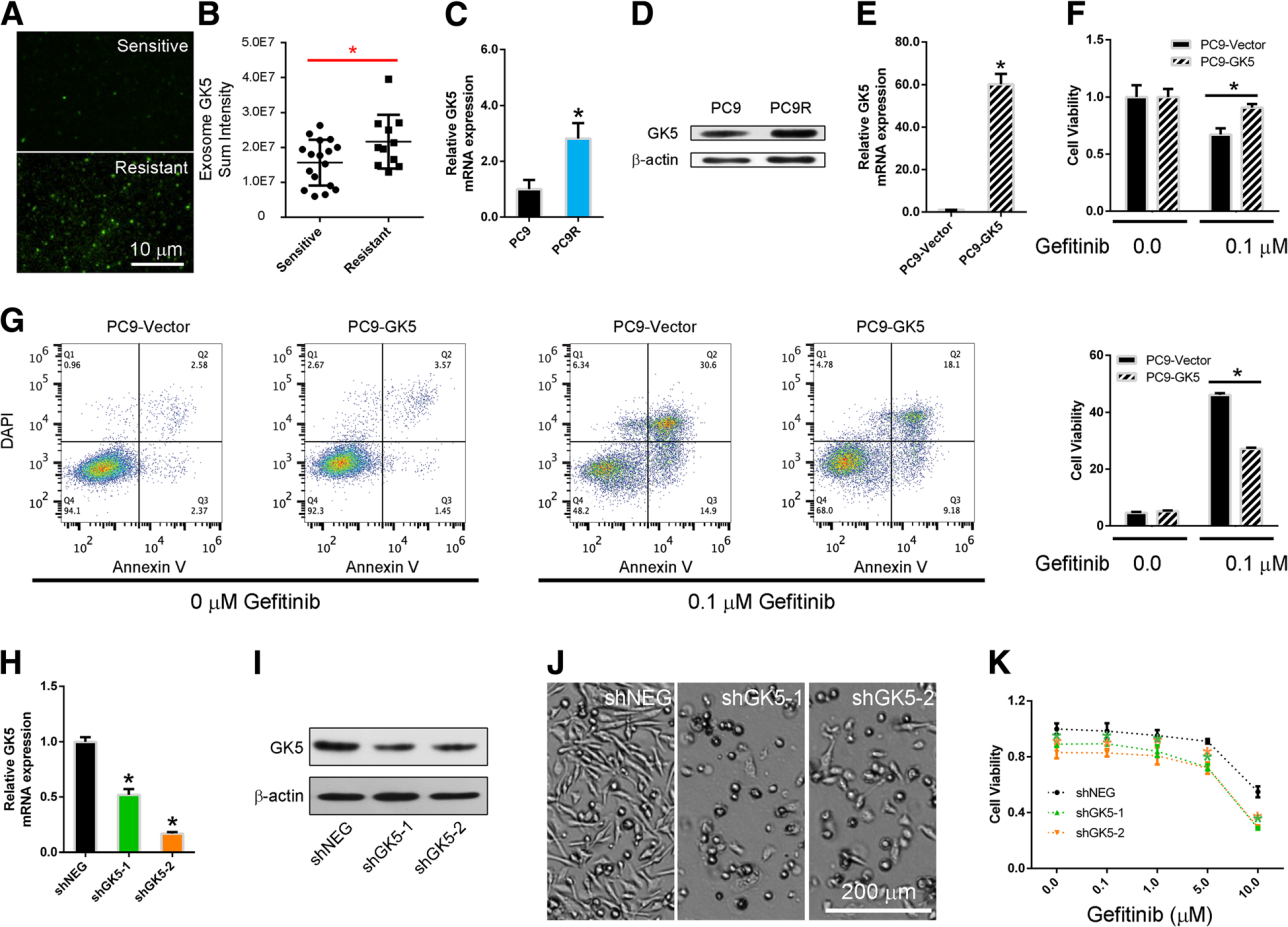
GK5 mediates gefitinib resistance. **a** Total Internal Reflection Fluorescence (TIRF) Microscopy images of exosomes isolated from plasma of EGFR TKI-sensitive and -resistant patients. **b** Exosomal GK5 mRNA in the plasma of gefitinib-sensitive and -resistant lung adenocarcinoma patients measured using TCLN biochips. **c**, **d** The RT-PCR and Western blotting on GK5 expression in gefitinib-sensitive PC9 and gefitinib-resistant PC9R cells. * *p* < 0.05 vs. the PC9 cells. **e** RT-PCR on GK5 expression in GK5-overexpressing (PC9-GK5) and control (PC9-Vector) PC9 cells. * *p* < 0.05 vs. control. **f**, **g** CCK8 assay and flow cytometry on viability and apoptosis of GK5-overexpressing and control PC9 cells. *P < 0.05 vs. control. **h**, **i** RT-PCR and Western blotting on GK5 expression in PC9R cells transfected with GK5-shRNA (shGK5–1 or − 2) or negative control shRNA (shNEG). **j** Morphology of PC9R cells expressing shGK5–1, − 2 or shNEG and cultured in the presence of 1 μM gefitinib. **k** CCK8 assay on the viability of PC9R cells expressing shGK5–1, − 2 or shNEG and treated with gefitinib. The data are representative of three experiments. * *p* < 0.05 vs. shNEG

To understand the association of GK5 expression and EGFR TKI resistance, we collected EDTA plasma samples from 28 lung adenocarcinoma patients, of which 17 and 11 were sensitive and resistant to EGFR-TKIs, respectively (Table 1). There were no differences in the clinical characteristics of these patients. Analysis of exosomal GK5 mRNA by a tethered cationic lipoplex nanoparticle based assay showed that the level of GK5 mRNA in plasma exosomes was downregulated in EGFR TKI-sensitive patients compared to patients who are TKI-resistant (Fig. 1a, b). To further understand the mechanism of gefitinib resistance, we generated gefitinib-resistant PC9R cells by exposing gefitinib-sensitive PC9 cells to increasing concentrations of gefitinib [39]. We found that in the gefitinib-resistant PC9R cells, GK5 was significant upregulated compared to that in gefitinib-sensitive PC9 by quantitative reverse transcription qRT-PCR and Western blotting analysis (Fig. 1c, d).

**Table 1:** The clinical characteristics of EGFR TKI-sensitive and -resistant lung adenocarcinoma patients

		Patient No.	EGFR TKI-sensitive	EGFR TKI-resistance	*P*
Total No.		28	17	11	
Age, median years		62.68	61.9	63.8	0.396
(range)		(59.5–65.8)	(58.2–65.7)	(57.4–70.2)	
Sex					0.315
	Female	16	11	5	
	Male	12	6	6	
Smoking					0.295
	Non-smokers	23	15	8	
	Smokers	5	2	3	
Stage					1
	IV	28	17	11

To evaluate the function of GK5 in gefitinib resistance, we constructed a lentivirus vector carrying the complete open reading frame of GK5 that allows GK5 overexpression in gefitinib-sensitive PC9 cells. After confirming GK5 overexpression in lentivirus-infected gefitinib-sensitive PC9 cells by qRT-PCR (Fig. 1e), we evaluated the viability and apoptosis of GK5-overexpressing and control cells by CCK8 assay and annexin V-APC/DAPI double staining followed by flow cytometry analysis, after treatment with gefitinib at concentrations ranging from 0 to 0.1 μM. GK5 induced drug resistance in gefitinib-sensitive PC9 cells (Fig. 1f, g), confirming that GK5 expression is sufficient to induce gefitinib resistance in cancer cells.

To further confirm the relationship between gefitinib-resistance and GK5 expression, gefitinib-resistant PC9R cells were transfected with either GK5 short hairpin (sh) RNA or negative control shRNA to silence GK5 expression. The qRT-PCR and Western blot analyses showed that shGK5–1 and − 2 significantly inhibited GK5 expressions (Fig. 1h, i). Morphological examination showed that GK5 knockdown reduced the number of PC9R cells transfected with shGK5 compared to those transfected with negative control shRNA for 24 h and then treated with 1 μM gefitinib for 72 h (Fig. 1j). The CCK8 assays revealed that transfection of PC9R cells with either shGK5–1 or − 2 enhanced gefitinib-induced apoptosis, especially at 10.0 μM gefitinib, increasing the percentage of apoptotic cells from 45 to 68 or 64%, respectively (Fig. 1k).

**Fig. 2:**
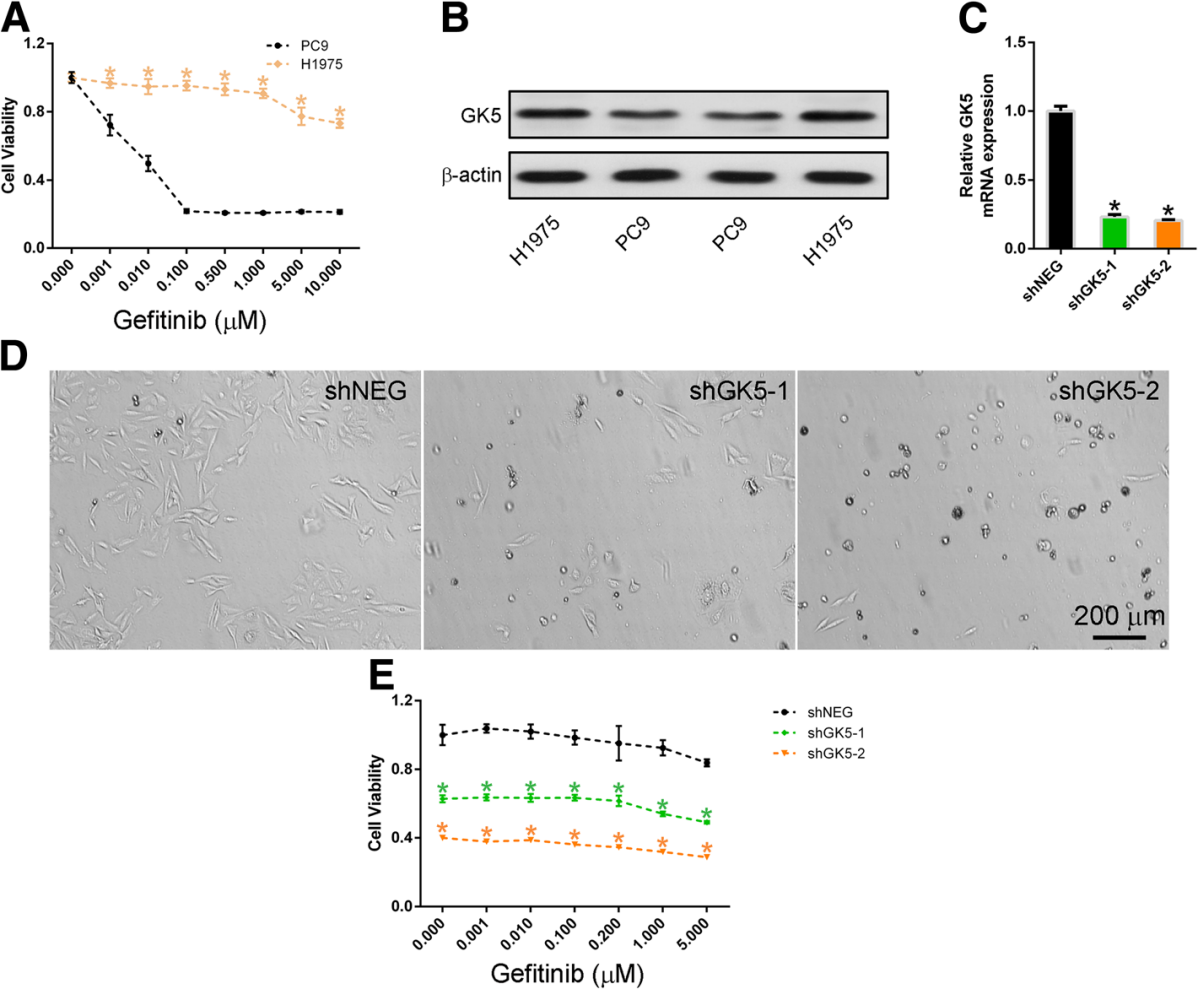
Knockdown of GK5 inhibits H1975 cell proliferation. **a** CCK8 assay on the gefitinib dose response in gefitinib-resistant H1975 and -sensitive PC9 cells. * *p* < 0.05 vs. the PC9 cells. **b** Western blotting on GK5 expression in gefitinib-sensitive PC9 and gefitinib-resistant H1975 cells. **c** RT-PCR on GK5 expression in H1975 cells transfected with GK5-shRNA (shGK5–1 or − 2) or negative control shRNA (shNEG). **d** Morphology of H1975 cells expressing shGK5–1, − 2 or shNEG and cultured in the presence of 1 μM gefitinib. **e** CCK8 assay on the viability of H1975 cells expressing shGK5–1, − 2 or shNEG and treated with gefitinib. The data are representative of three experiments. * *p* < 0.05 vs. shNEG

To verify the relationship between gefitinib-resistance and GK5 expression, gefitinib-resistant H1975 cells, which contain the exon 20 T790 M mutation, were used. To validate gefitinib resistance in H1975 cells, the viability of PC9 and H1975 cells upon gefitinib treatment was measured by CCK-8 assay. It was found that the viability of the PC9 cells was dramatically reduced in the presence of 0.1 μM gefitinib, whereas the viability of the H1975 cells was not significantly decreased by up to 1 μM gefitinib treatment (Fig. 2a). Western blotting demonstrated that the protein level of GK5 was upregulated in gefitinib-resistant H1975 cells compared to PC9 cells (Fig. 2b). The qRT-PCR analyses showed that shGK5–1 and − 2 significantly inhibited GK5 expressions in H1975 cells (Fig. 2c). Morphological examination showed that GK5 knockdown reduced the number of H1975 cells transfected with shGK5 compared to those transfected with negative control shRNA for 24 h and then treated with 1 μM gefitinib for 72 h (Fig. 2d). The CCK8 assays revealed that transfection of H1975 cells with either shGK5–1 or − 2 enhanced gefitinib-induced apoptosis (Fig. 2e).

### GK5 knockdown induces PC9R cell apoptosis and cell cycle arrest in the presence of gefitinib

**Fig. 3:**
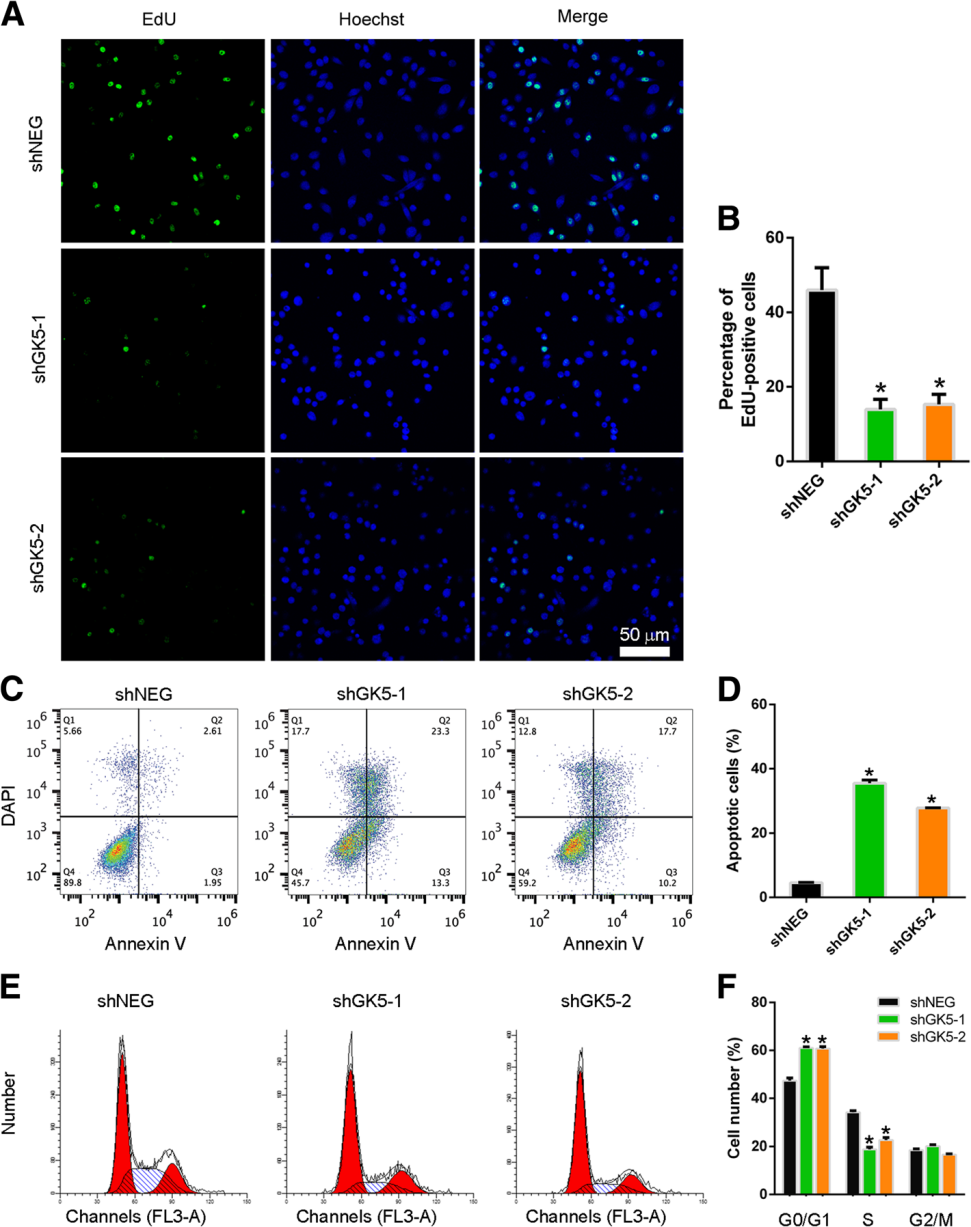
Knockdown of GK5 inhibits PC9R cell proliferation, increases apoptosis, and induces cell cycle arrest. **a** PC9R cells expressing GK5-shRNA (shGK5–1 or − 2) or negative control shRNA (shNEG) were treated with 1 μM gefitinib and then stained with EdU and Hoechst 33342. **b** The percentage of EdU-positive cells was higher in the control group than in the GK5-silenced groups. **c** Flow cytometry on PC9R cells expressing shGK5–1, − 2 or shNEG. Cells treated with 1 μM gefitinib and stained with APC-annexin V/DAPI. **d** GK5 silencing increased apoptosis in PC9R cells. **e** Cell cycle analyzed by flow cytometry. **f** Flow cytometry on cell cycle of PC9R cells expressing shGK5–1, − 2 or shNEG. Cells were treated with 1 μM gefitinib, and the number of cells in different phases of cell cycle was detected. The data are representative of three experiments. * p < 0.05 vs. shNEG

To confirm that GK5 knockdown suppresses the proliferation of gefitinib-resistant cells in the presence of gefitinib, we used 5-ethynyl-2′-deoxyuridine (EdU) staining to analyze the effect of GK5 silence on proliferation of gefitinib-resistant cells following gefitinib treatment. The fraction of EdU-positive cells was significantly lower in GK5-silenced cells compared with control cells, indicating a lower rate of cell proliferation in the absence of GK5 (Fig. 3a, b). We also analyzed the apoptosis by annexin-V-APC and DAPI double staining followed by flow cytometry. The results showed that cell apoptosis increased ~ 35% in PC9R cells transfected with shGK5 relative to negative control cells in the presence of 1 μM gefitinib (Fig. 3c, d). Analysis of cell cycle distribution by flow cytometry showed that GK5 silencing resulted in cell number increased in the G0/G1 phase and decreased in S phase in the presence of 1 μM gefitinib, indicating that cell cycle arrest was induced by GK5 knockdown (Fig. 3e, f).

### GK5 knockdown induced PC9R cell mitochondrial dysfunction and caspase activation

**Fig. 4:**
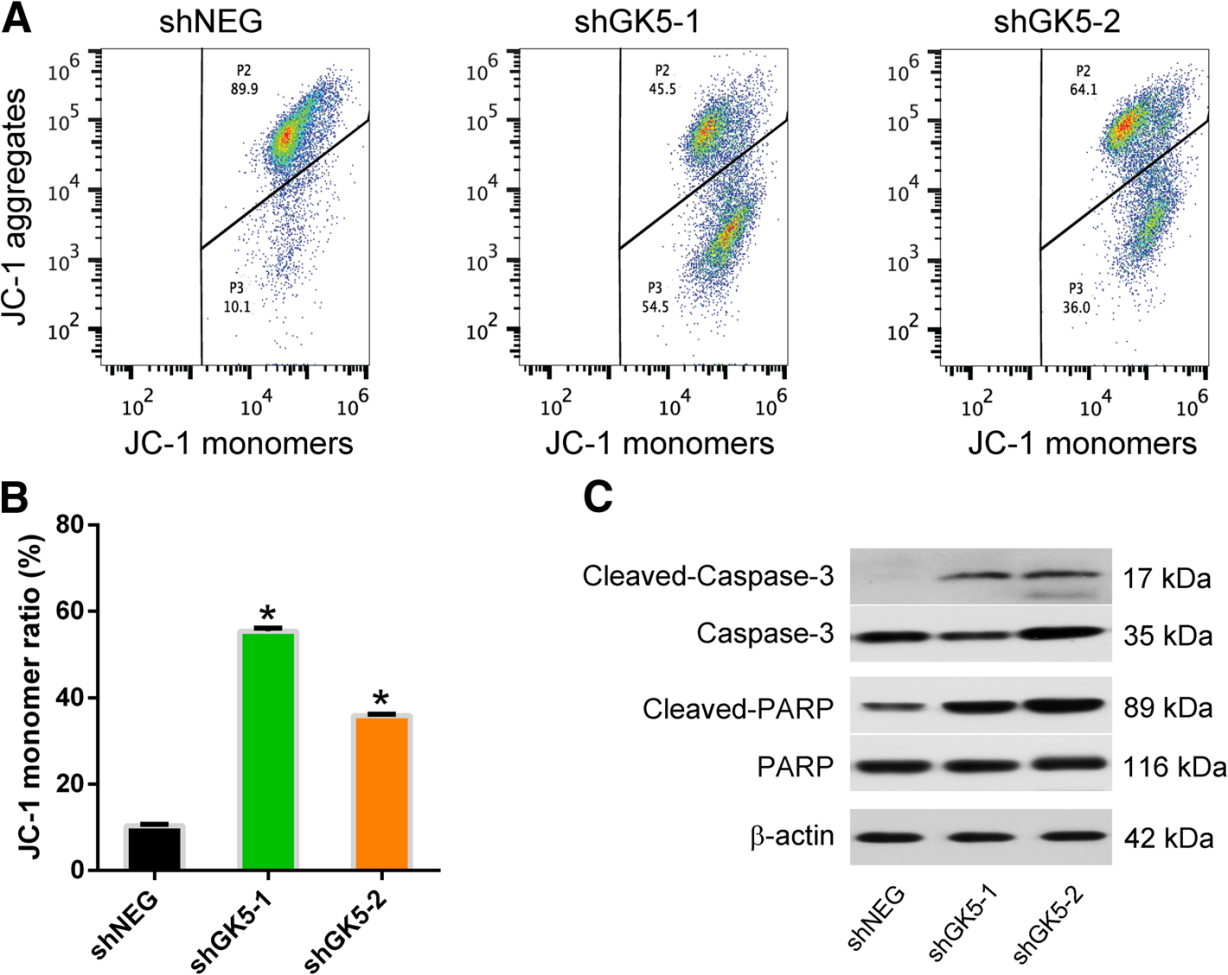
Downregulation of GK5 decreases mitochondrial membrane potential. **a** Representative histograms showing the flow cytometry analysis on JC-1 staining. **b** The statistical analysis on the JC-1 monomer ratio in PC9R cells compared to negative control cells incubated with 1 μM gefitinib. The data are representative of three experiments. * p < 0.05 vs. shNEG. **c** Western blot analysis of cleaved caspase-3, total caspase-3, cleaved PARP and total PARP protein in PC9R cells transfected with shGK5–1, − 2 or shNEG

We examined mitochondrial membrane potential of PC9R cells transfected with shGK5 or negative control shRNA. Using JC-1 staining, which emits red fluorescence as J-aggregates in intact mitochondria and green fluorescence in the monomeric form in damaged mitochondria. The JC-1 monomer ratio of PC9R cells transfected with shGK5 was higher than that of cells transfected with the negative control in the presence of 1 μM gefitinib, indicating that the loss of GK5 expression was associated with mitochondrial damage (Fig. 4a, b). Mitochondria dysfunction can accelerate caspase-3 activation, as evidenced by Western blotting showing increased amounts of cleaved caspase-3 and poly-ADP-ribose polymerase (PARP) in GK5-depleted cells compared to control cells (Fig. 4c).

### GK5 knockdown suppresses tumor proliferation in vivo

**Fig. 5:**
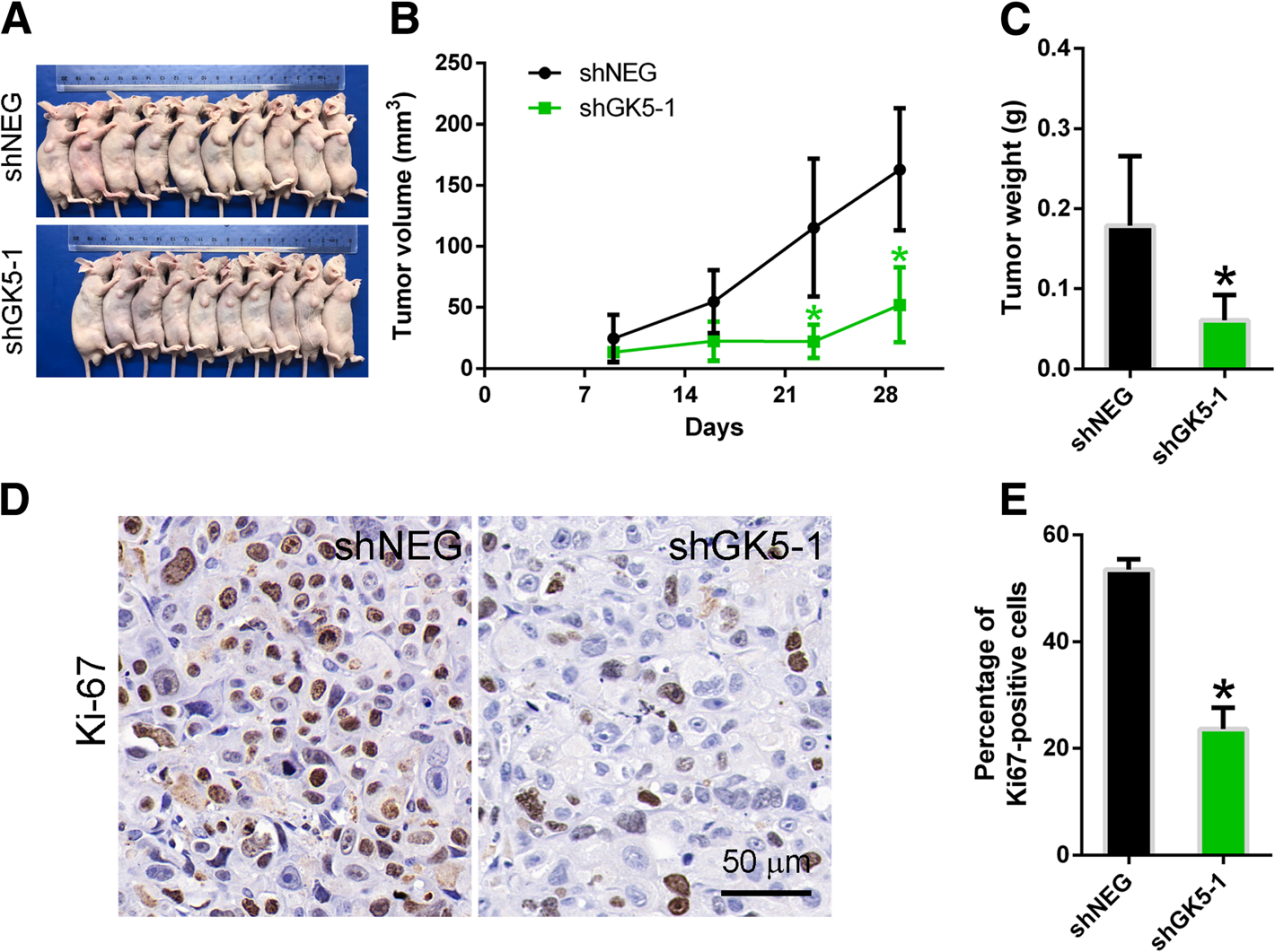
GK5 knockdown suppresses tumor cell proliferation in vivo. **a** Nude mice with tumors one month after injection of PC9R cells expressing GK5-shRNA (shGK5–1) or negative control (shNEG). **b** Tumor volume measured once a week. **c** Quantitative analysis on tumor weight of shGK5–1 or shNEG groups. **d** Ki67 expression in xenograft tumors. **e** Quantitative analysis of Ki67-positive cells. *N* = 10 mice per group. * p < 0.05 vs. shNEG

To determine whether GK5 knockdown suppresses lung tumor growth in vivo, PC9R cells transfected with shGK5–1 or negative control shRNA were injected into nude mice. PC9R cells transfected with shGK5–1 or negative control shRNA grew into visible tumors one week after injection, although the size of tumors derived from GK5-silenced cells was significantly smaller than that of tumors from control cells (Fig. 5a). A similar trend in tumor mass was seen one month after injection (Fig. 5b, c). To assess the effects of GK5 knockdown on cell proliferation, Ki67 expression in sections of tumors was detected by immunohistochemistry. The percentage of Ki67-positive cells decreased in tumors derived from GK5-silenced cells compared with tumors from control cells (Fig. 5d, e).

### GK5 confers gefitinib resistance through upregulating SCD1 expression

**Fig. 6:**
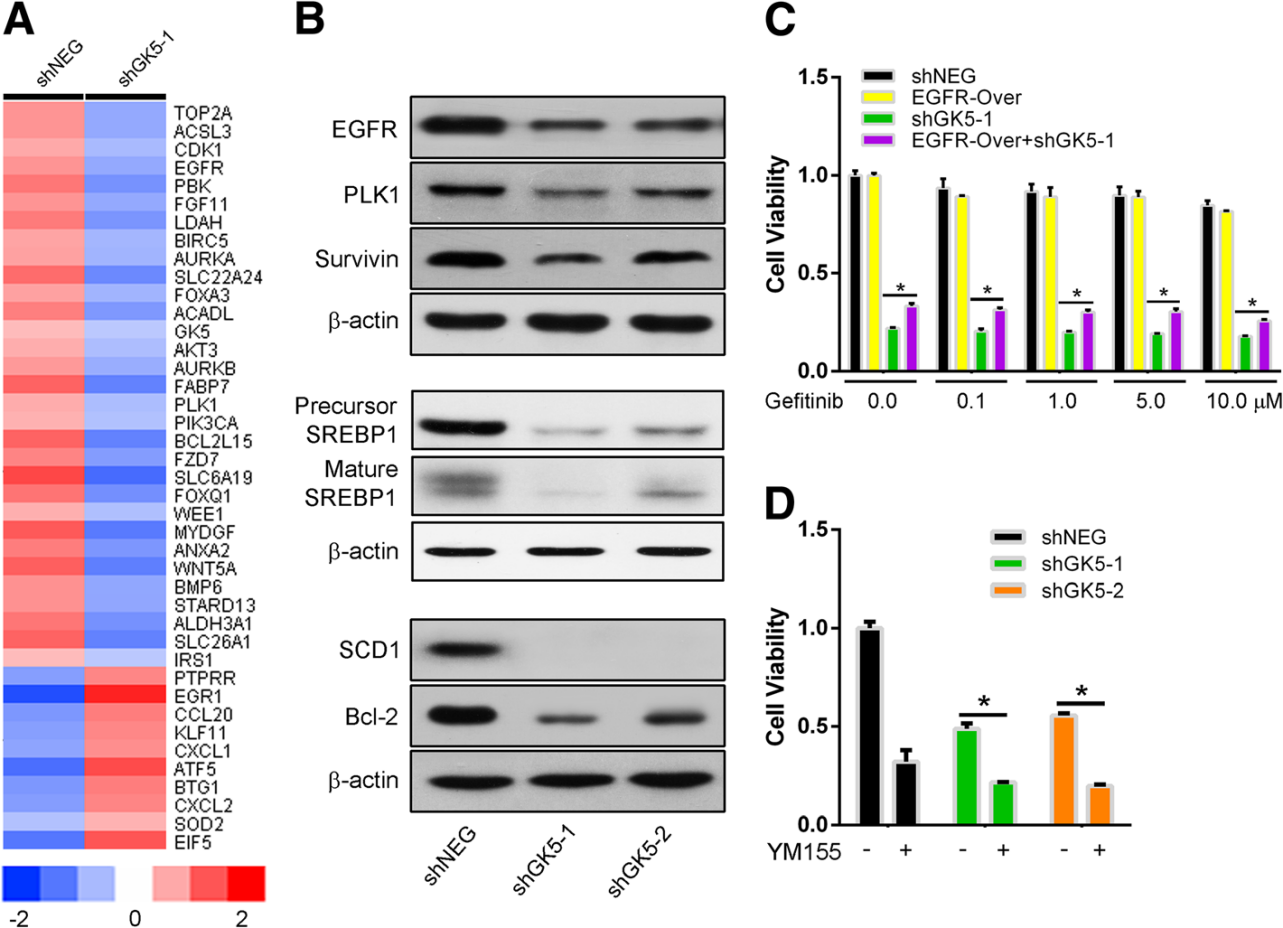
GK5 knockdown suppresses PC9R cell proliferation by inhibiting EGFR/AKT/SREBP1/SCD1 signaling molecules. **a** Whole human cDNA array analysis. **b** Western blotting on protein levels of EGFR, p-AKT, AKT, survivin, PLK1, SREBP1, SCD1 and Bcl-2 in PC9R cells **c** CCK8 assay on cell proliferation of PC9R cells overexpressing EGFR and infected with viral particles expressing shGK5–1 and shNEG. **d** CCK8 assay on cell proliferation of PC9R cells transfected with shGK5–1, − 2 or shNEG and treated with YM155. * *p* < 0.05 vs. shNEG

To evaluate the molecular mechanism underlying GK5-mediated gefitinib resistance, we used a whole human cDNA array to identify alterations in GK5 signaling pathways. This assay showed that GK5 depletion altered the expression of metabolic, apoptosis and cell cycle-associated molecules (Fig. 6a). Western blotting confirmed that GK5 knockdown in gefitinib-resistant PC9R cells significantly repressed protein expression of SREBP1 and SCD1 (Fig. 6b). The expression of EGFR, Survivin, PLK1, and Bcl-2 were also repressed.

To confirm the relationship between GK5 and EGFR, we performed a rescue assay by overexpressing EGFR in PC9R cells. CCK8 assays showed that EGFR overexpression in PC9R cells partly rescued the apoptotic effects of GK5 depletion in PC9R cells after treatment with gefitinib (Fig. 6c). YM155, a potent survivin inhibitor, also decreased the proliferation of PC9R cells relative to the negative control, especially in cells transfected with shGK5, indicating that GK5 induces drug resistance by interfering survivin signaling (Fig. 6d).

**Fig. 7:**
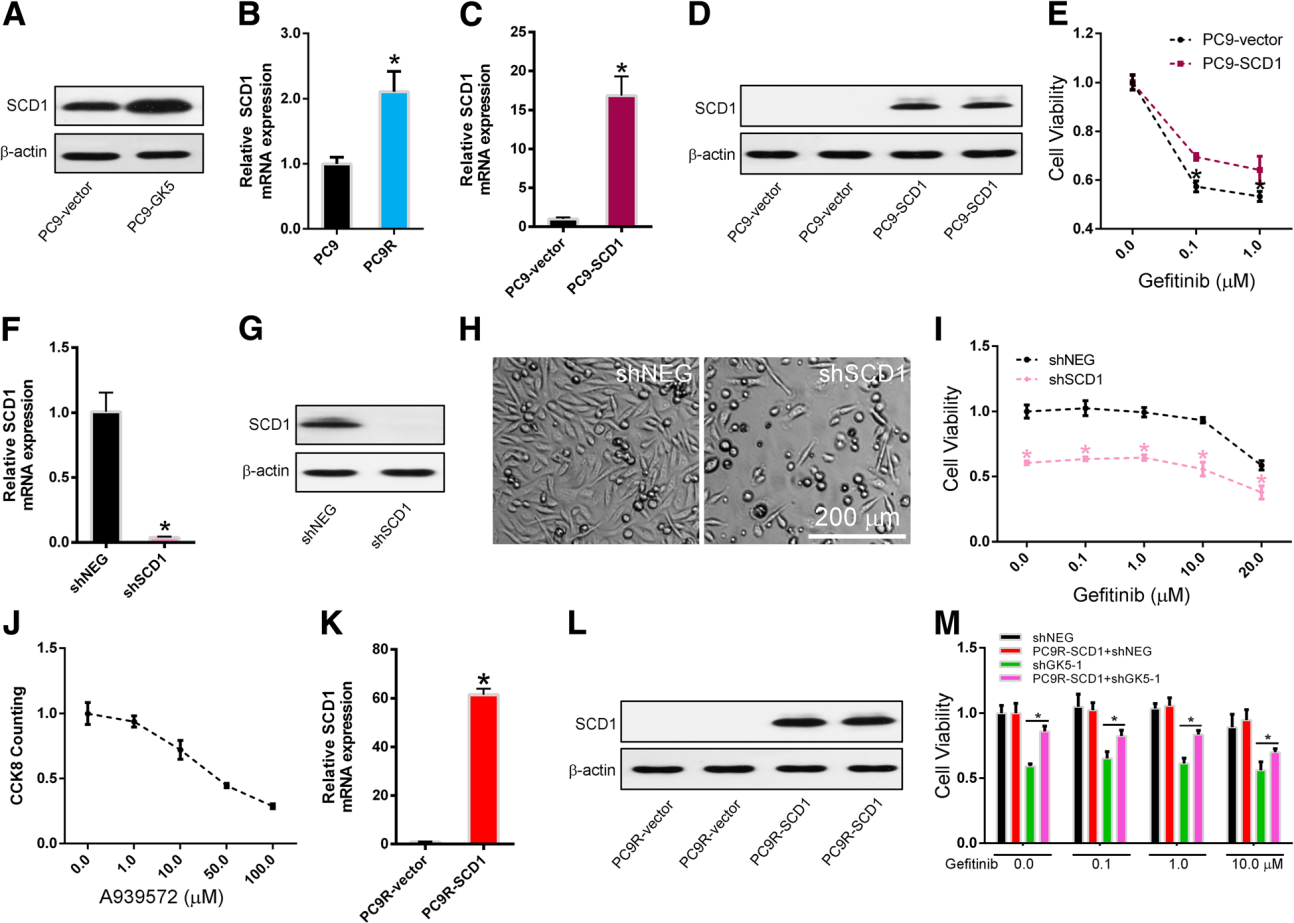
SCD1 confers gefitinib resistance. **a** Western blotting on SCD1 expression in GK5-overexpressing (PC9-GK5) and control (PC9-Vector) PC9 cells. **b** RT-PCR on SCD1 expression in gefitinib-sensitive PC9 and gefitinib-resistant PC9R cells. * p < 0.05 vs. PC9 cells. **c**, **d** RT-PCR and Western blotting on SCD1 expression in SCD1-overexpressing (PC9-SCD1) and control (PC9-Vector) PC9 cells. * p < 0.05 vs. control. **e** CCK8 assay on cell viability of SCD1-overexpressing and control PC9 cells treated with gefitinib. *P < 0.05 vs. control. **f**, **g** RT-PCR and Western blot analysis of SCD1 expression in PC9R cells transfected with SCD1-shRNA (shSCD1) or negative control shRNA (shNEG). **h** Morphology of PC9R cells expressing shSCD1 or shNEG and cultured in the presence of 1 μM gefitinib. **i** CCK8 assay on cell viability of PC9R cells expressing shSCD1 or shNEG and treated with gefitinib. The data are representative of three similar experiments. * p < 0.05 vs. shNEG. **j** CCK8 assay on cell viability of PC9R cells treated with SCD1 inhibitor A939572. **k**, **l** RT-PCR and Western blot on SCD1 expression in SCD1-overexpressing (PC9R-SCD1) and control (PC9R-Vector) PC9R cells. * *p* < 0.05 vs. control. **m** CCK8 assay on cell proliferation of PC9R cells overexpressing SCD1 and infected with viral particles expressing shGK5–1 and shNEG. * *p* < 0.05 vs. shNEG

Furthermore, we found that SCD1 was upregulated in GK5 overexpressed PC9 cells (Fig. 7a). We also found that SCD1 was significantly increased in the PC9R cells compared with PC9 cells (Fig. 7b). We constructed a lentivirus vector carrying the complete open reading frame of SCD1 that allows SCD1 overexpression in PC9 cells. After confirming SCD1 overexpression in lentivirus-infected gefitinib-sensitive PC9 cells by qRT-PCR and Western blot (Fig. 7c, d), we evaluated the viability of SCD1-overexpressing and control cells by CCK8 assay after treatment with gefitinib. We found that SCD1 induced drug resistance in gefitinib-sensitive PC9 cells (Fig. 7e), confirming that SCD1 expression is sufficient to induce gefitinib resistance in cancer cells.

To further confirm the relationship between gefitinib-resistance and SCD1, PC9R cells were transfected with either SCD1 short hairpin (sh) RNA or negative control shRNA to silence SCD1 expression. The qRT-PCR and Western blotting showed that shSCD1 significantly inhibited SCD1 expressions (Fig. 7f, g). Morphological examination showed that SCD1 knockdown reduced the number of PC9R cells transfected with shSCD1 compared to those transfected with negative control shRNA for 24 h and then treated with 1 μM gefitinib for 72 h (Fig. 7h). To verify this, the CCK8 assay was performed and revealed that transfection of PC9R cells with shSCD1 enhanced gefitinib-induced apoptosis (Fig. 7i). Furthermore, A939572, a specific SCD1 inhibitor, decreased the proliferation of PC9R cells in a dose-dependent manner (Fig. 7j).

Then we constructed a lentivirus vector carrying the complete open reading frame of SCD1 that allows SCD1 overexpression in PC9R cells. After confirming SCD1 overexpression in lentivirus-infected gefitinib-sensitive PC9R cells by qRT-PCR and Western blot (Fig. 7k, l), a rescue assay was performed in the SCD1-overexpressed PC9R cells. CCK8 assays showed that SCD1 overexpression in PC9R cells transfected with shGK5 cells rescued PC9R cell viability in the presence of gefitinib (Fig. 7m). These results suggest that SCD1 overexpression decreased cell apoptosis induced by GK5 depletion.

## Discussion

EGFR-TKIs have become the first-line treatment for advanced NSCLC that carry EGFR mutations [40]. However, about 20% of NSCLC patients carrying EGFR mutation do not respond to EGFR-TKIs, and most NSCLC patients that initially benefited from EGFR-TKIs develop resistance [41]. Thus, identifying novel therapeutic targets associated with TKI resistance is an urgent need. In this study, we showed that GK5 is upregulated in gefitinib-resistant clinical samples and lung cancer cells, and mediates gefitinib resistance. Overexpression of GK5 further increased gefitinib resistance, whereas knockdown of GK5 expression reduced gefitinib resistance by promoting cell apoptosis and cycle arrest through activation of the EGFR/SREBP1/SCD1 signaling pathway.

In recent years, metabolic pathways such as Warburg effect and lipogenesis that drive cancer proliferation and resistance to drug are extensively studied [10, 42]. GK5 is an important factor in glycerol and triglyceride metabolism [33, 36]. However, the expression and function of GK5 in EGFR-TKI resistance of NSCLC is not clear. Here we show that exosomal GK5 mRNA in the plasma from gefitinib-resistant adenocarcinoma patients was significantly higher compared to gefitinib-sensitive patients. Furthermore, GK5 was overexpressed in gefitinib-resistant cells, indicating that GK5 could play a role in gefitinib resistance. Indeed, GK5 knockdown induced apoptosis of gefitinib-resistant PC9 cells by impairing mitochondrial function.

Genes controlling lipogenesis, including SREBP1, FASN, SCD1, and acetyl-CoA carboxylase-1 (ACC1), are frequently upregulated in cancer cells [41]. Silencing these genes significantly reduce proliferation and induce apoptosis in cancer cells [43]. In consistent with this finding, lipogenesis inhibitors inhibit cell viability and increase cell apoptosis in a number of cancers [10]. Thus, targeting components of lipid metabolism could overcome resistance in cancer therapeutics [10]. Here we confirm that SCD1 is overexpressed in gefitinib-resistant cells. Overexpression of SCD1 increased gefitinib resistance, whereas genetic ablation of SCD1 expression reduced gefitinib resistance by promoting apoptosis and cell cycle arrest. Furthermore, A939572, an inhibitor of SCD1, significantly decreases the viability of gefitinib-resistant PC9R cells. We further show that GK5 knockdown significantly inhibit the protein levels of SREBP1 and SCD1, indicating that GK5 might regulate cell proliferation and gefitinib resistance via SREBP1/SCD1 signaling pathway.

EGFR is frequently found to be overexpressed in various cancers, and resistance to EGFR TKIs is associated with EGFR overexpression [44]. EGFR has both tyrosine kinase dependent and independent functions [25, 29, 45], both of which activate downstream signalling pathways such as PI3K/Akt and are essential for cell proliferation [23]. In gefitinib-resistant cells, EGFR tyrosine kinase-dependent pathway does not respond to gefitinib. Thus, targeting EGFR tyrosine kinase-independent pathway becomes an alternative strategy to overcome gefitinib resistance. Here we show that silencing GK5 significantly inhibits the protein level of EGFR, and overexpression of EGFR in PC9R cells partly rescued the apoptotic effects of GK5 depletion, indicating that GK5 induces drug resistance by interfering EGFR signaling.

EGFR has been demonstrated to be involved in Warburg effect and lipogenesis [13, 22, 29]. EGFR modulates glucose levels in cancer cells by regulating sodium/glucose cotransporter 1 (SGLT1) independent of receptor tyrosine kinase activities [29], and activates SREBP1 and SCD1 through PI3K/Akt pathway [13, 22], indicating that GK5 might allow cancer cells to escape apoptosis promoted by gefitinib treatment by regulating EGFR/PI3K/AKT/SREBP1/SCD1 pathway.

Taken together, our results suggest that exosomal GK5 mRNA might be used as a biomarker in gefitinib resistance, and silencing GK5 might reverse the acquired resistance. GK5 could be a potential target for treating patients with EGFR-TKI-resistant lung cancer.

## Conclusions

We demonstrated that GK5 confers gefitinib resistance in lung cancer by inhibiting apoptosis and cell cycle arrest. GK5 could be a novel therapeutic target for treatment of NSCLC with resistance to EGFR tyrosine kinase inhibitors.

## Abbreviations

ACC: Acetyl-coa carboxylase
ACC1: Acetyl-CoA carboxylase-1
APC: Annexin-V-allophycocyanin
ATCC: American Type Culture Collection
CCK8: Cell counting kit-8
DAPI: 4′,6-diamidino-2-phenylindole
EdU: 5-ethynyl-2′-deoxyuridine
EGFR: Epidermal growth factor receptor
FAM: Fluorescein amidite
FASN: Fatty acid synthase
GK5: Glycerol kinase 5
MB: Molecular beacon
NSCLC: Non-small cell lung cancer
p-AKT: phosphorylated AKT
PARP: Poly-ADP-ribose polymerase
p-ERK: phosphorylated extracellular signal-regulated kinase
PI3K: Phosphoinositide 3-kinase
qRT-PCR: Quantitative Real-time PCR
SCD1: Stearoyl-CoA desaturase-1
SGLT1: Sodium/glucose cotransporter 1
shRNA: Short hairpin RNA
SREBP1: Sterol regulatory element-binding protein 1
TCLN: Tethered cationic lipoplex nanoparticle
TIRF: Total Internal Reflection Fluorescence
TKI: Tyrosine kinase inhibitor
